# Cervical Microbiome in Women Infected with HPV16 and High-Risk HPVs

**DOI:** 10.3390/ijerph192214716

**Published:** 2022-11-09

**Authors:** Thanayod Sasivimolrattana, Wasun Chantratita, Insee Sensorn, Arkom Chaiwongkot, Shina Oranratanaphan, Pattarasinee Bhattarakosol, Parvapan Bhattarakosol

**Affiliations:** 1 Medical Microbiology Interdisciplinary Program, Graduate School, Chulalongkorn University, Bangkok 10330, Thailand; 2 Center of Excellence in Applied Medical Virology, Department of Microbiology, Faculty of Medicine, Chulalongkorn University, Bangkok 10330, Thailand; 3 Center for Medical Genomics, Faculty of Medicine, Ramathibodi Hospital, Mahidol University, Bangkok 10400, Thailand; 4 Division of Virology, Department of Microbiology, Faculty of Medicine, Chulalongkorn University, Bangkok 10330, Thailand; 5 Department of Obstetrics and Gynecology, Faculty of Medicine, Chulalongkorn University, Bangkok 10330, Thailand; 6 Department of Mathematics and Computer Science, Faculty of Science, Chulalongkorn University, Bangkok 10330, Thailand

**Keywords:** cervical cancer, microbiome, bacterial community, fungal community, viral community, human papillomavirus, HPV

## Abstract

Human papillomavirus type 16 (HPV16) and/or high-risk (Hr-) HPV are the main causes of cervical cancer. Another element that may contribute to the development of cervical cancer is the microbiota. To date, no study has investigated the entire cervical microbiome, which consists of bacteria, fungi, and viruses. In this study, cervical samples with different histopathology (CIN1, CIN2, and CIN3), with or without HPV16 and Hr-HPVs infection, were enrolled. From bacterial community analysis, 115 bacterial species were found and separated into 2 distinct categories based on *Lactobacillus* abundance: Lactobacilli-dominated (LD) and non-Lactobacilli-dominated (NLD) groups. The LD group had significantly less bacterial diversity than the NLD group. In addition, the variety of bacteria was contingent on the prevalence of HPV infection. Among distinct histological groups, an abundance of *L. iners* (>60% of total *Lactobacillus* spp.) was discovered in both groups. A few fungi, e.g., *C. albicans*, were identified in the fungal community. The viral community analysis revealed that the presence of HPV considerably reduced the diversity of human viruses. Taken together, when we analyzed all our results collectively, we discovered that HPV infection was a significant determinant in the diversity of bacteria and human viruses in the cervix.

## 1. Introduction

Cervical cancer is one of the most prevalent malignancies in women worldwide and the second most prevalent disease in emerging nations [1]. More than 90% of cervical cancer cases are caused by HPV infection [2]. HPV is transmitted through direct skin contact. Although the HPV vaccine has >90% protective efficacy [3,4], it cannot prevent women who have had prior HPV infections [5].

HPV is a member of the *Papillomaviridae* family, which is currently split into 16 genera, of which 5 genera contain members that infect humans, namely *Alpha-*, *Beta*-, *Gamma*-, *Mupa*-, and *Nupapapillomavirus* [6]. Over 200 distinct kinds of HPV have been found [7]. Based on the likelihood of cancer development, HPV is categorized into two primary groups: “high-risk HPVs” (Hr-HPVs) and “low-risk HPVs” (Lr-HPVs). Hr-HPVs include types 16, 18, 31, 33, etc. [8], while Lr-risk HPVs include genotypes 6, 11, etc. HPV16 is the most prevalent cause of cervical cancer [6]. Lr-HPVs typically cause benign hyperproliferative lesions, such as condyloma acuminatum (CA), and infrequently induce malignant cancer in the genital tract [9]; nevertheless, malignancy at the larynx may be observed [10,11]. 

Although HPV infection is the primary cause of cervical cancer, environmental factors may also play a significant part in the disease’s progression. The International Agency for Research on Cancer (IARC) identified smoking as a co-factor of cervical cancer in 2004 [12,13]. Other elements, such as the microbiological environment, were understudied. In 2014, Hung et al. shown that vaginal microbial communities serve crucial roles in maintaining vaginal homeostasis and preventing the infection of harmful bacteria [14]. *Lactobacilli* are believed to have a crucial function in vaginal health [15]. One or two species of *Lactobacillus* are often the most common microorganisms found in a healthy vagina [16,17]. The vagina microbiota has been divided into five major community state types based on the dominance of *Lactobacillus* spp., namely CST I (dominated by *L. crispatus*), CST II (dominated by *L. gasseri*), CST III (dominated by *L. iners*), CST IV (dominated by a high diversity of bacterial vaginosis (BV)-related bacteria), and CST V (dominated by *L. jensenii*) [16]. These *Lactobacillus* spp. promote a protective environment in the vagina by producing metabolites, bacteriocins, hydrogen peroxide (H_2_O_2_), and lactic acid, and by lowering the pH by producing hydrogen peroxide [18,19,20,21]. Observations include not only bacterial but also fungal populations. More than 70% of vaginal fungus are *Candida albicans*. Typically, this *C. albicans* acts as an opportunistic pathogen [22]. Nevertheless, the predominance of microbial communities differs amongst ethnic groups [16,23,24].

A few studies have detected, in addition to vaginal microbiota, the microbiota in the cervix where HPV infection occurs. Klein et al. [25] demonstrated that the bacterial diversity of HPV-negative cervical samples is much lower than that of HPV-positive samples. In addition, a positive link is established between bacterial abundance and the stage of HPV-associated cervical cancer development (CIN3 > CIN1 patients) in the vagina and cervix [26]. Not only is bacterial diversity greater in Hr-HPV-infected samples than in Lr-HPV-infected samples, but only Hr-HPVs-infected patients possess *Malassezia* sp. [26]. Viruses can also function as a microbial community in numerous human body regions [27]. Several viruses, such as papillomavirus, alphatorquevirus, and anellovirus, were detected by metagenomic investigation of viruses in the healthy vagina [28]. Moreover, the investigation of the human viral community in the cervix of HIV/HPV co-infected patients revealed the presence of several viruses from the *Papillomaviridae*, *Anelloviridae*, *Genomoviridae*, and *Herpesviridae* families [29]. Intriguing would be a systematic research of the microorganisms present in the HPV16- and Hr-HPV-positive cervix of women with various lesion abnormalities. This study intends to investigate the bacterial, fungal, and viral communities in the cervix of Thai patients infected with HPV16 and Hr-HPV at various precancerous stages.

## 2. Materials and Methods

### 2.1. Sample Recruitment

The specimens were taken by colposcopy from patients who visited the Department of Gynecological Outpatient at King Chulalongkorn Memorial Hospital in Bangkok, Thailand. During a colposcopy, a gentle swab at the posterior fornix of the cervix using an Ayre Spatula was performed by a gynecologist. The swab was resuspended in lysis buffer and the sample was then rapidly stored at −80 °C until use. The Cobas 4800 high-risk HPV test identified the presence of HPV16, 18, and Hr-HPVs DNA in all samples at the Virology Unit, Department of Microbiology, King Chulalongkorn Memorial Hospital in Bangkok, Thailand (Roche, Basel, Switzerland). Blood-free samples with either HPV16 or Hr-HPVs positivity and reproductive-age women (21–50 years old) with histopathology results were the inclusion criteria for the selected samples (CIN1, CIN2, or CIN3). Basically, the classification of cervical intraepithelial neoplasia (CIN) ranges from 1 to 3. CIN1 refers to abnormal cells that compose around one-third of the epithelium’s thickness. Meanwhile, about two-thirds and more than two-thirds of the epithelium thickness are abnormal cells, known as CIN2 and CIN3, respectively. Exclusion criteria were menstruation at the time of specimen collection, antibiotic or vaginal antimicrobial usage during the previous month, current microbiological infection, HIV infection, and pregnancy. Only 43 of the remaining HPV-positive specimens met these requirements. Moreover, five CIN1 with HPV-negative samples were selected as a control group.

### 2.2. Total Nucleic Acid Extraction from Cervical Specimens

The COBAS 4800 instrument automatically extracted total nucleic acid from the remaining cervical specimens (Roche, Basel, Switzerland). Prior to DNA extraction, the sample was beaten at 30 Hz for 10 min using Pathogen Lysis Tubes L (Qiagen, Hilden, Germany) and TissueLyser LT (Qiagen, Hilden, Germany) [30]. 

### 2.3. The Study of the Bacterial and Fungal Communities

The Swift amplicon 16S + ITS panel amplified the 16S rRNA of bacteria and ITS gene of fungus (Swift Biosciences, Ann Arbor, MI, USA). This kit included a single pool of primers for all variable sections of the 16S rRNA gene (V1–V9) plus the fungal ITS1 and ITS2 genes. Based on a library size of 475 bp, the libraries were quantified using the KAPA library quantification kit (Roche, Basel, Switzerland).

Before sequencing, libraries were denatured and diluted in accordance with the instructions for the MiSeq System (document number 15039740 v10; Illumina, San Diego, CA, USA). The library was adjusted to 11 pM by HT1 and placed onto the MiSq Reagent Kit v2 reagent cartridge (Illumina, San Diego, CA, USA). On an Illumina MiSeq platform, sequencing was then performed to generate two 151 bp reads.

The Illumina Miseq FASTQ data were exported for the bacterial analysis. The Microbial Genomics Module of QIAGEN CLC Genomics Workbench 20 (version 20.0.4) was used to examine the sequences. The database utilized was Greengenes v13.8. At least 10 distinct reads were valid. In the case of any atypical microorganism, the sequence reads were reanalyzed by BLAST against the NCBI taxonomy database using 16S ribosomal RNA sequences (Bacteria and Archea) as the database. At least 97% and 99% sequence identity were acceptable for genus- and species-level identifications, respectively [31]. The data were analyzed using the ITS Metagenomics Module version 1.1.0 on BaseSpace Sequence Hub (Illumina) for the Fungal community analysis. The algorithm and database utilized were the high-performance implementation of the Ribosomal Database Project (RDP) Classifier [32] and UNITE v7.2 [33], respectively. Any OTUs in any sample with fewer than 10 readings were removed from further analysis.

### 2.4. The Study of the Viral Community

The cDNA and the second strand were generated from the extracted total nucleic acid to recover RNA viruses from samples. cDNA was produced using Roche Evoscript Universal cDNA Master and random hexamers from an RNA template (Roche, Basel, Switzerland). Second-strand buffer (10× NEB Buffer 2), 0.7 mM dNTP mix, 0.18 U/L RNaseH, 0.7 U/L DNA polymerase I, and 1.8 mM DTT were then utilized to synthesize the second strand of cDNA (New England Biolabs, Ipswich, MA, USA).

Slight modifications were made to the SeqCap EZ Hypercap Workflow Version 2.3 (Roche, Basel, Switzerland) recommendations to prepare the gDNA collection. Each reagent was acquired from Roche (Basel, Switzerland). gDNA was sheared enzymatically with the KAPA HyperPlus Library Preparation Kit. In brief, the gDNA was combined with KAPA Frag Buffer/Enzyme and incubated at 37 °C for 15 min to obtain an average fragment size of 200 bp. NanoDrop (Eppendorf, Hamburg, Germany) was used to ensure the quality and yield of the above libraries. The Fragment Analyzer 3700 with PRO Size 3.0 software (Agilent, Santa Clara, CA, USA) was utilized to examine the size distribution, which must be between 150 and 500 bp with a peak of 320 bp.

The samples were hybridized with the SeqCap EZ probe pool (VirCapSeq-VERT Capture Panel), which contains 2.1 million probes (varying lengths of 50–105 mers) encompassing the genomes of 207 viral species [34]. NanoDrop yield and purity measurements were used to assess the quality of each library. Using the Fragment Analyzer 3700, the size distribution was studied. To check potential contamination, library preparation with a negative control (water) was also performed; however, the fragment size of this negative control following library preparation could not be determined.

### 2.5. Illumina Sequencing and Data Analysis for the Viral Community

The libraries were quantitated by the KAPA library quantification kit. The sample preparation was carried out by using the denature and dilute libraries guide for the MiSeq System (Document # 15039740 v10; Illumina, San Diego, CA, USA) using the MiSeq Reagent Kit v3 (Illumina, San Diego, CA, USA). The procedure followed the reagent leaflet. The library was adjusted to 18 pM by HT1 and loaded onto the reagent cartridge for sequencing using an Illumina MiSeq platform to generate 2 × 300 bp reads.

The NGS raw FASTQ files were explored for the presence of viruses in each sample. The Virus Identification Pipeline (VIP) was run on Docker and Ubuntu 20.04 LTS and was performed by using sense mode [35]. In the quality-control step, low-quality and adapter reads were trimmed while the low-complexity sequences were removed by using the DUST algorithm. The trimmed reads of more than 20 bp were retained by using PRINSEQ [36]. After that, the host-related reads were subtracted by using Bowtie2. Then, the bacteria and related rRNA (ribosomal RNA) reads were discarded, and the remaining reads were aligned to the virus database. Unmatched reads were aligned to a viral protein database from NCBI Refseq DB using RAPSearch. Finally, all matched reads were classified under a genus for de novo assembly and phylogenetic analysis. All virus databases were downloaded from ftp://ftp.ncbi.nih.gov/refseq/release/viral/ (accessed on 11 October 2020).

### 2.6. Statistics

The hierarchical clustering tree was generated using UPGMA and Euclidean as the algorithm and similarity index, respectively, via Qiagen CLC Genomics Workbench 20 (Version 20.0.4) using the Microbial Genomics Module (bacterial community) and Past 4.03 software (viral community). The heat map was constructed by CLC software (bacterial community) and GraphPad Prism 8 software (fungal and viral communities). The alpha (Shannon, total number of species, and Chao1 richness estimator) and beta diversity (principal coordinate analysis (PCoA) based on Bray–Curtis dissimilarities of the microbial community structure) were calculated by CLC (bacterial community), ITS Metagenomics Module (fungal community), and Past 4.03 software (viral community), whereas the boxplots, rarefaction curve, bar chart, and pie chart were constructed by GraphPad Prism 8 software. To observe the statistical difference between the alpha diversity and richness, a non-parametric unpaired *t*-test (Mann–Whitney test) was performed, whereas PERMANOVA was used for beta diversity analysis. A Chi-square test was used to calculate the similarity of percent ubiquity among samples.

## 3. Results

### 3.1. Characteristics of Clinical Samples

A total of 48 (43 HPV-positive and 5 HPV-negative) leftover cervical samples were collected. They were classified according on the results of histology, i.e., cervical intraepithelial neoplasia (CIN) type 1 and CIN2/3 (Table S1). Due to the extremely uncommon instances of HPV negativity in the cervix of women undergoing colposcopic evaluation, only five CIN1 samples with HPV negativity were discovered and used as a control group. The mean age of patients in each group was displayed (Table S1). All 48 enrolled patients had mean ± SEM ages of 37.77 ± 1.25 years (ranging from 23 to 50 years old). There was no significant difference in age among the histological groups (*p =* 0.1688, CIN1 HPV-negative vs. CIN1; *p =* 0.2701, CIN1 HPV-negative vs. CIN2/3; *p =* 0.5187, CIN1 vs. CIN2/3; Mann–Whitney U-test). The peripheral clinical data of the patients are shown in Table S2.

### 3.2. Cervical Bacterial Community in Association to Histological Characteristics and HPV Infection

**Figure 1:**
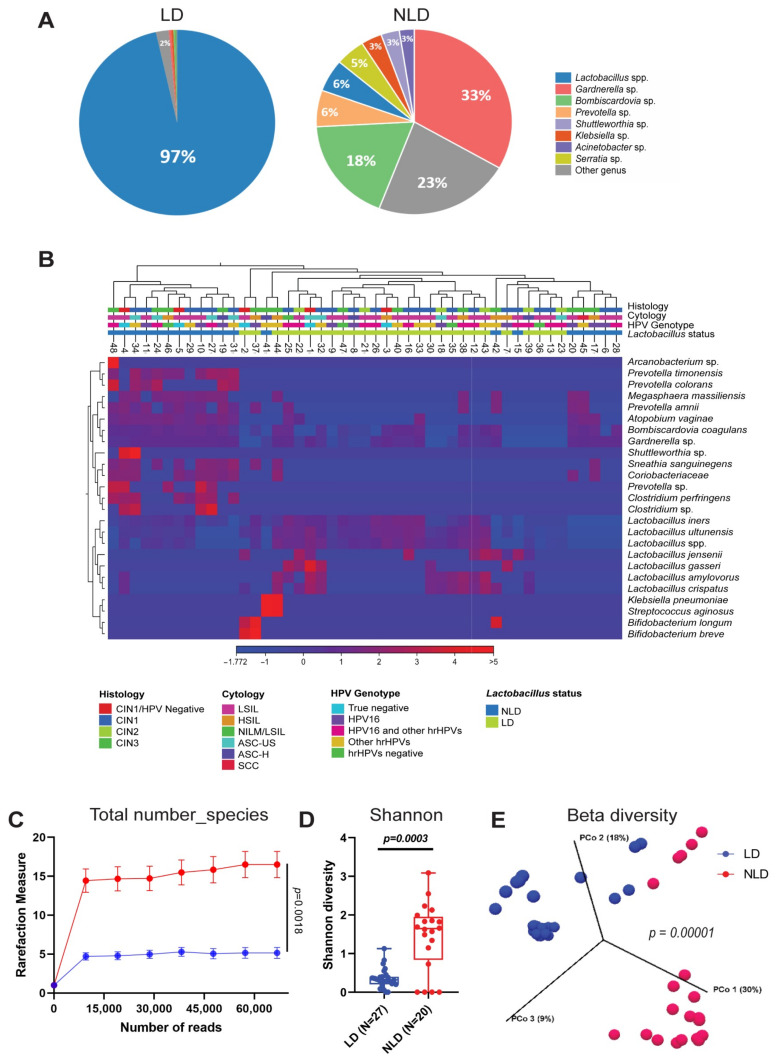
The composition of bacterial community in cervical samples. (**A**) The percentage of the top 8 bacterial genera found in the cervix. (**B**) The hierarchical clustering tree and heat map of bacterial taxa were generated using UPGMA and Euclidean as the algorithm and similarity index, respectively. The color key under the tree represents histology, cytology, HPV genotypes, and Lactobacillus status. Blue to red color shades represent increasing relative abundance. (**C**) α-diversity, total number of species, (**D**) α-diversity, Shannon diversity index, and (**E**) β-diversity. Error bars represent the standard error of the mean (SEM). Blue = LD, Red = NLD.

The Illumina MiSeq raw data for all 48 cervical samples were exported. One sample lacked sufficient nucleic acid for analysis, so only forty-seven samples were included in the study. A total of 3.7 M (3,793,991) high-quality reads were retrieved with an average of 80,723 per sample. One hundred and fifteen species of bacteria were found in cervical samples. *Lactobacillus* spp., *Gardnerella* sp., *Bombiscardovia* sp., *Prevotella* sp., and *Shuttleworthia* sp. were the five taxa that predominated in the cervical samples (Figure S1A). Lactobacilli-dominated (LD; *n* = 27) and non-lactobacilli-dominated (NLD; *n* = 20) cervical microbiota groups were identified based on the 60% abundance of *Lactobacillus* spp. [37]. In the LD and NLD groups, the average relative abundance of *Lactobacillus* spp. was 97% and 6%, respectively (Figure 1A). The LD group had numerous *Lactobacillus* species, including *L. iners*, *L. crispatus*, and *L. amylovorus*. In contrast, *Lactobacillus* spp. were exceedingly rare in the NLD group, although other bacteria, such as *Gardnerella* spp., *Bombiscadovia coagulans*, and *Atopobium vaginae*, were prevalent (Figure 1B). In both groups, *L. iners* was the predominant *Lactobacillus* species, followed by *L. crispatus* for LD and *L. jensenii* for NLD (Figure S1B,C). The alpha diversity was substantially greater in the NLD group than in the LD group (Figure 1C,D and Supplementary Figure S2A). Similar outcomes were reported with beta diversity (Figure 1E).

**Figure 2:**
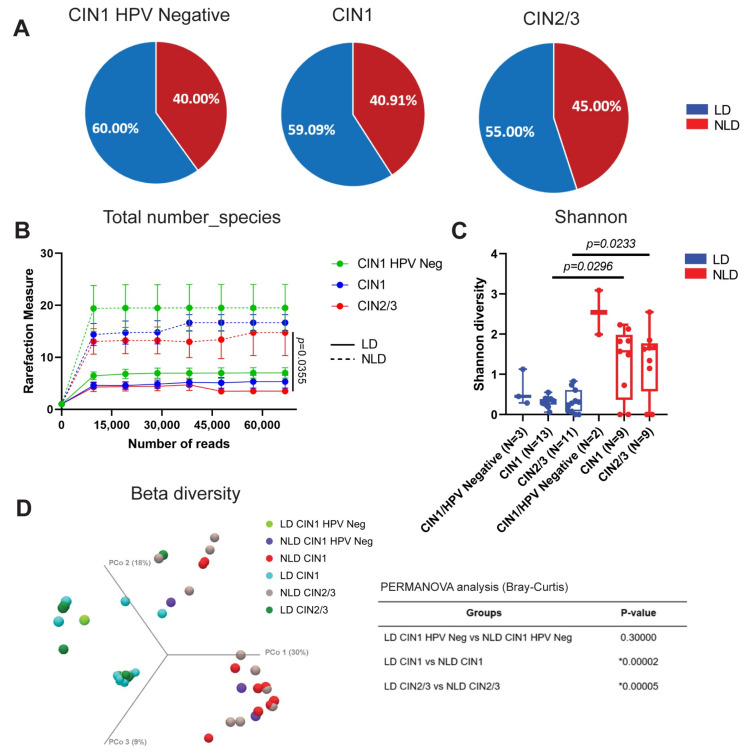
The relationship between bacterial diversity and histological characteristics. (**A**) Pie chart represents the percentage of LD and NLD in different histological groups. (**B**) α-diversity, total number of species, (**C**) α-diversity, Shannon diversity index, and (**D**) β-diversity. Error bars represent the standard error of the mean (SEM). Asterisk indicates statistically significant difference by PERMANOVA analysis (Bray-Curtis).

In this investigation, samples were separated into three categories: CIN1 HPV-negative, CIN1, and CIN2/3. CIN1 HPV-negative (40.00%) and CIN1 (40.091%) had comparable percentages of NLD samples, however CIN2/3 had a slightly greater percentage of NLD samples (45%) (Figure 2A). The alpha diversity based on the total number of species (Figure 2B) and the Shannon index (Figure 2C) was statistically distinct between the pairs of LD and NLD in CIN1 and CIN2/3, but not in the pair of CIN1 HPV-negative. These outcomes were identical to alpha diversity as measured by the Chao1 index (Figure S2B). Similar to the results of alpha diversity analysis, beta diversity analyses revealed significant changes in bacterial community structure between groups (Figure 2D).

**Figure 3:**
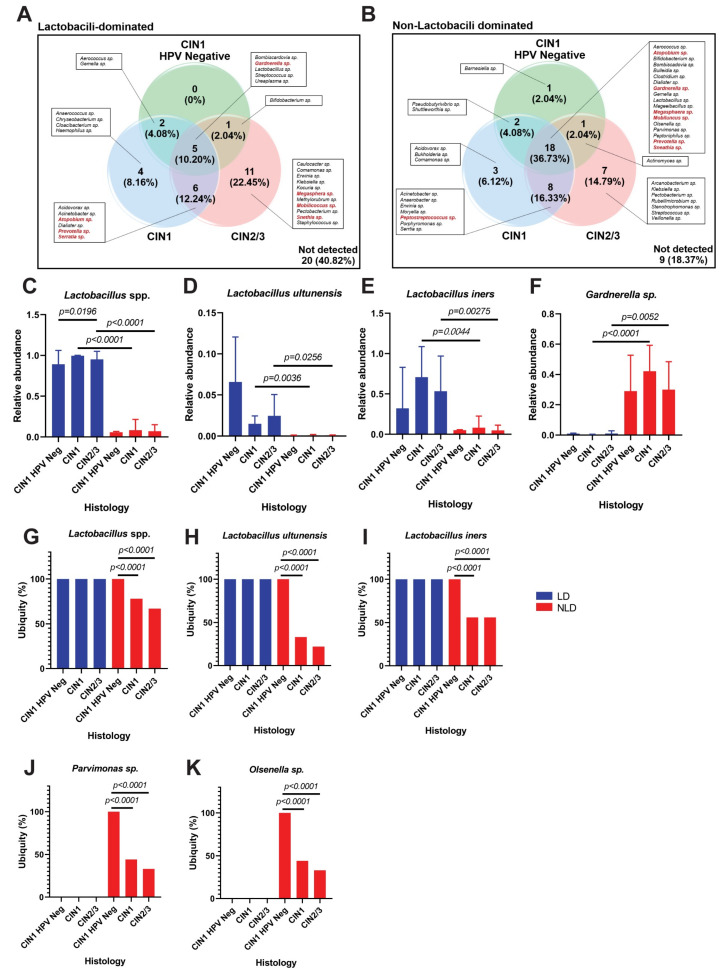
The unique and shared bacterial taxa in the cervical samples. Venn diagram represents numbers of unique and shared operating taxonomy unit (OTU) at the genus level in different histological groups: LD (**A**) and NLD (**B**). Red highlight represents the genus of bacteria involved in bacterial vaginosis (BV). Relative abundance of the selected taxa: *Lactobacillus* spp. (**C**), *L. ultunensis* (**D**), *L. iners* (**E**), and *Gardnerella* sp. (**F**). Mann–Whitney U-test for non-parametric data was used to compare the abundance between each group. Error bars represent the standard error of the mean (SEM). The ubiquity (%) of the selected taxa: *Lactobacillus* spp. (**G**), *L. ultunensis* (**H**), *L. iners* (**I**), *Parvimonas* sp. (**J**), and *Olsnella* sp. (**K**). Chi-Square test was used to compare the ubiquity between groups.

The number of unique genera in CIN1 HPV-negative, CIN1, CIN2/3, and shared between groups of LD and NLD is demonstrated in Figure 3A,B. In the LD group, 5 genera (10.20%) were shared in all histological groups (Figure 3A). In the NLD group, up to 18 genera (36.73%) were common in all histological groups, for example, *Atopobium* sp., *Bifidobacterium* sp., *Bombiscadovia* sp., *Gardnerella* sp., *Prevotella* sp., *Sneathia* sp., and *Lactobacillus* spp. (Figure 3B). Significant abundances of *Lactobacillus* spp., *L. ultunesis*, *L. iners*, and *Gardnerella* sp. were demonstrated in LD both in CIN1 and CIN2/3 when compared to NLD groups. No significant difference was found between CIN1 HPV-negative LD and NLD (Figure 3C–F). The differences in relative abundance of other bacterial species were established in Table S3. All LD groups had 100% ubiquity of *L. ultunensis* and *L. iners*, whereas the percentage of ubiquity in the NLD group decreased significantly as the disease severity increased (Figure 3G–I). Interestingly, *Parvimonas* sp. and *Olsnella* sp. were detected only in the NLD group with 100% ubiquity in CIN1 HPV-negative and significantly higher than HPV-positive groups (Figure 3J,K). The differences in ubiquity of other bacterial species are provided in Table S4.

### 3.3. Cervical Fungal Community in Association to Histological Characteristics and HPV Infection

**Figure 4:**
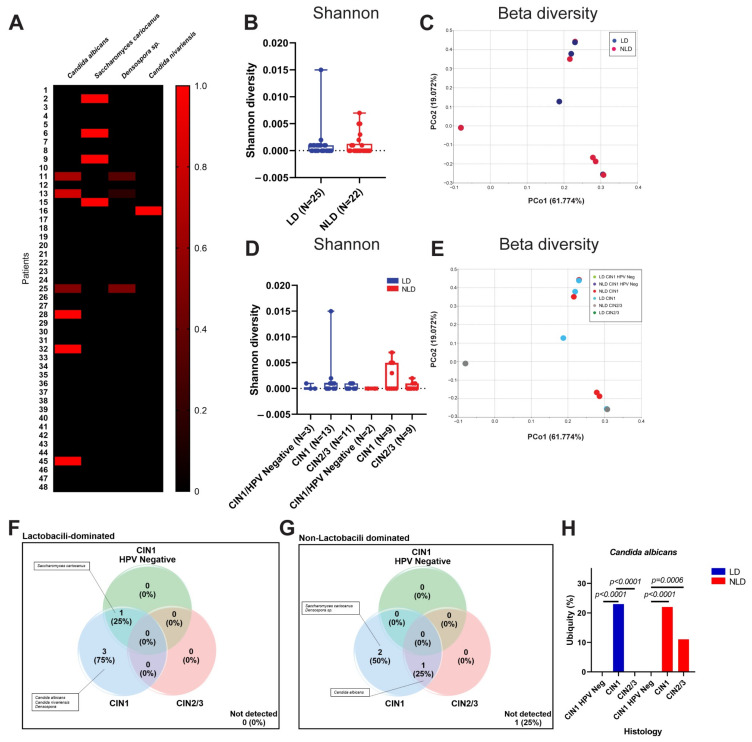
The composition and diversity of the fungal community in cervical samples. (**A**) Heat map of fungal taxa from cervical samples. Black to red color shades represent increasing relative abundance. The fungal diversity between LD and NLD groups: alpha (Shannon) diversity (**B**) and beta diversity (**C**). Fungal diversity among different histological groups: alpha (Shannon) diversity (**D**) and beta diversity (**E**). Error bars represent the standard error of the mean (SEM). Venn diagram represents numbers of unique and shared fungal OTU in different histological groups: LD (**F**) and NLD (**G**). The ubiquity (%) of the selected taxa: *Candida albicans* (**H**).

The 3.7 M of high-quality 16S/ITS reads were analyzed by BaseSpace Sequence Hub through the ITS Metagenomics Module. Only four taxa of the fungi were found, including *Candida albicans*, *C. nivariensis*, *Saccharomyces cariocanus*, and *Densospora* sp. (Figure 4A). Neither LD nor NLD nor histological groups showed a significant difference in fungal diversity (Figure 4B–E). The unique and shared fungal taxa among all sample groups are shown in Figure 4F,G. There was no significant difference in the relative abundance of any fungi across the groups (Table S5). *C. albicans* was not detectable in both LD and NLD CIN1 HPV-negative samples, but only in NLD CIN1, LD CIN1, and LD CIN2/3 samples, with a statistically significant difference (Figure 4H and Table S5).

### 3.4. Cervical Viral Community in Association to Histological Characteristics and HPV Infection

Due to the low concentration of input total nucleic acid, data were only acquired from 35 of the 48 samples in this study. A total of 8.5 M (8,562,489) high-quality reads were retrieved. Using VIP, 1.5 M (1,514,093) viral reads were subtracted [35]. The mean number of viral reads per sample was 43,260. Interestingly, numerous species of viruses were discovered in cervical samples. Figure S3 reveals the twenty most common virus species. HPV16 was the most dominant viral species. The percent coverage of the HPV genome per sample ranged from 24.27% to 100%, with an average of 79.85%. In addition to being human viruses (HPV), the viruses found in these cervical samples were specific to different hosts, including insect virus, protozoa virus, bird virus, and mammalian virus (Figure S3).

**Figure 5:**
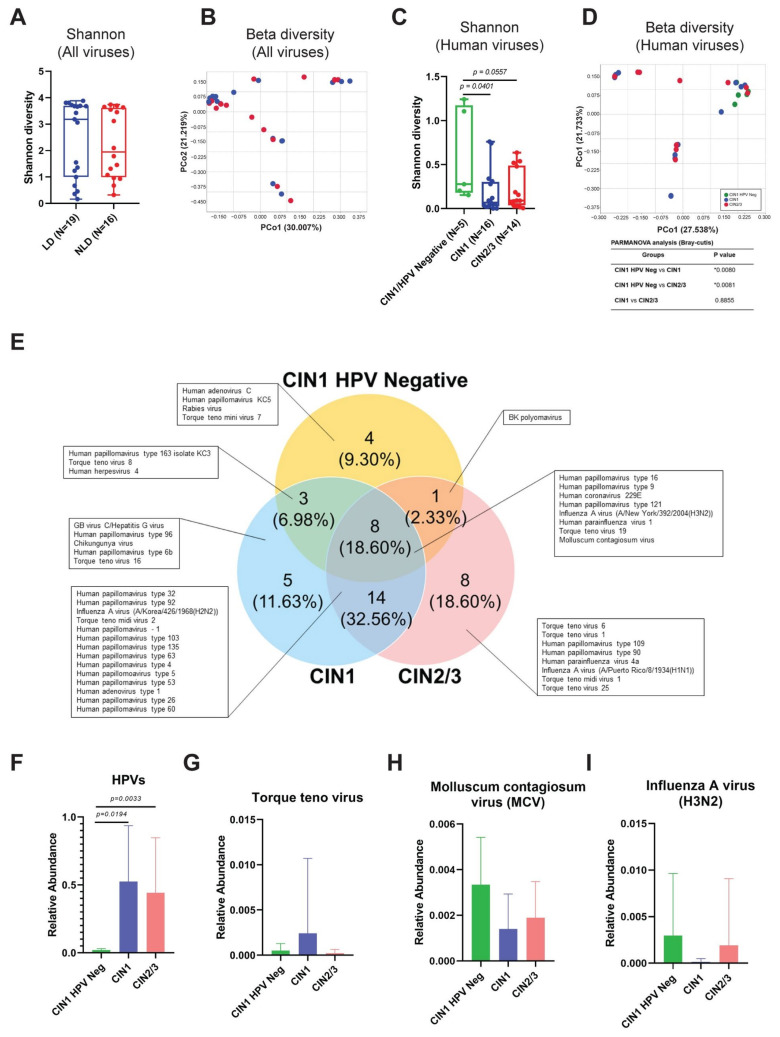
The composition of the viral community in cervical samples. (**A**,**B**) The viral diversity between LD (blue) and NLD (red) groups. (**C**,**D**) Viral diversity of Human viruses among different histological groups: Shannon diversity (alpha diversity) (**A**,**C**) and beta diversity (**B**,**D**). Asterisk indicates statistically significant difference by PERMANOVA analysis (Bray-Curtis). (**E**) Venn diagram represents the unique and shared viral OTU (species) among histological groups. (**F**–**I**) Relative abundance of each selected viral specie in all groups. Mann–Whitney U-test for non-parametric data was used for comparisons between the abundance of each group. Error bars represent the standard error of the mean (SEM).

To examine the link between *Lactobacillus* spp. dominance and the viral community, alpha and beta diversity were computed. The viral diversity between LD and NLD groups did not differ significantly (Figure 5A,B). Due to the multiplicity of observed virus–host groups, we concentrated exclusively on the variety of human viruses. Surprisingly, the presence of HPV greatly decreased the diversity of human viruses (Figure 5C,D).

Eight species of human viruses (18.60%) were shared by all histological categories (Figure 5E), however certain viral species were exclusive to CIN1 HPV-negative, CIN1, and CIN2/3, such as Human adenovirus C, Hepatitis G virus, and Influenza A virus (H1N1), respectively. In addition, many types of Torque teno viruses (TTVs) were detected in many groups. Unexpectedly, a few HPV16 reads were detected in all CIN1 HPV-negative individuals (Figure 5F). Of these 5 samples, 3 were LD, whereas 2 were NLD. By exporting the reads and assembling them with the reference sequence, CLC Genomics Workbench 22 validated the existence of HPV16 in the five CIN1 HPV-negative groups. Then, at least three distinct sequences were blasted against the NCBI using the nucleotide collection (nr/rt) as the database. Nonetheless, the relative abundance of HPVs in HPV-positive CIN1 and CIN2/3 was considerably greater than in HPV-negative CIN1. The abundance of TTVs, Molluscum contagiosum virus (MCV), and Influenza A virus (H3N2) did not change significantly between groups (Figure 5G–I). The ubiquity of Influenza A virus (H2N2) was detected in CIN1 and CIN2/3 but not in the CIN1 HPV-negative group (Figure S4), but the ubiquity of other viruses, including Influenza A virus (H3N2) and TTVs, was comparable.

**Figure 6:**
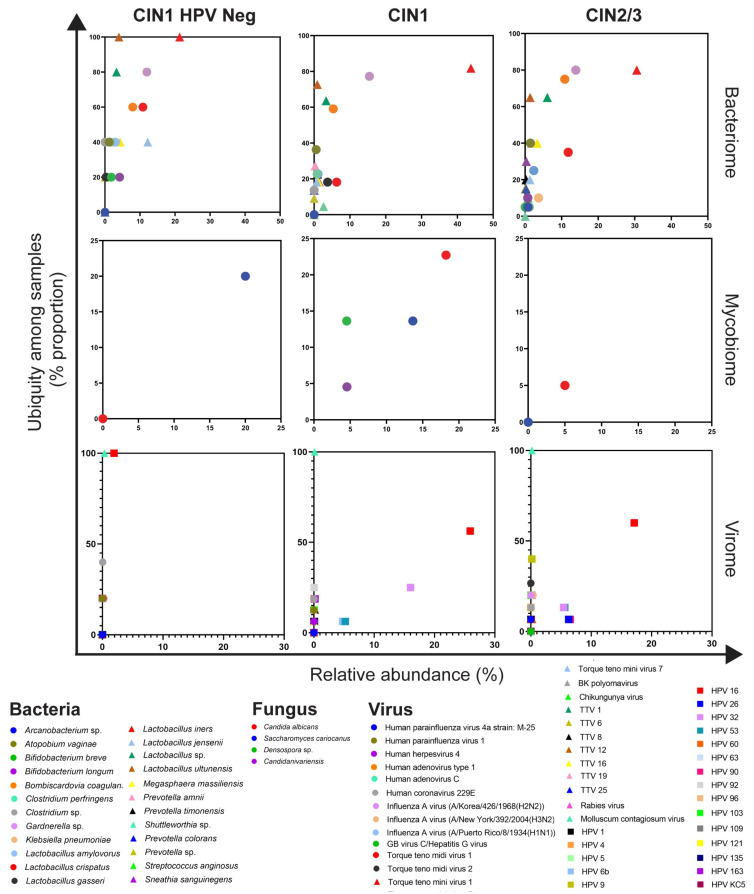
Ubiquity dot plot representing bacteria, fungi, and virus OTUs in the cervix with different histological characteristics. The graph was plotted between the mean of ubiquity (*y* axis) and relative abundance (*x* axis) of each OTU.

The abundance and ubiquity of bacteria, fungi, and viruses found in the cervix of women with varying degrees of dysplasia were analyzed. *L. iners* was the most prevalent bacterial taxon in all groups (Figure 6). Although *L. iners* was discovered in the CIN1 HPV-negative group (100%), its relative abundance was higher in CIN1 (43.78%) and CIN2/3 (30.54%) than in CIN1 HPV-negative (21.33%). *Gardnerella* sp. was the second most common bacterial taxa in all groups. *C. albicans* was absent in the CIN1 HPV-negative group, although it was present in the CIN1 and CIN2/3 groups. As expected, the HPV16 abundance was the most predominant virus in all groups. However, the relative abundance of HPV16 in CIN1 HPV-negative samples was low (1.93%) compared to HPV-positive samples (CIN1 25.93%, CIN2/3 17.96%). Besides HPV16, other viruses were discovered. Notably, MCV was found in all samples (Figure 6).

## 4. Discussion

Despite that the vaginal microbiome has been demonstrated for several years, the microbial composition of the vagina has been found to differ between ethnic groups [16,23,24]. Besides the vagina, the cervical microbiome was significantly different [38]. Consequently, the purpose of this study was to investigate the bacterial, fungal, and viral communities in the cervix of HPV16- and Hr-HPV-infected patients. According to our findings, the cervical bacterial community of Thai patients infected with HPV could be divided into LD and NLD groups. Compared to the NLD group, the LD group had a very low level of cervical bacterial diversity (Figure 1C,D). This might be due to *Lactobacillus* spp., which often generate bacteriocin and a low pH to inhibit the growth of bacterial vaginosis (BV)-associated bacterial species [39]. *Lactobacillus* spp. acidic pH generation hindered the colonization of harmful bacterial species, such as *Chlamydia trachomatis* and *Gardnerella vaginalis* [39,40]. This state was necessary to protect the cervix’s epithelial barrier because BV-associated bacteria can create enzymes and metabolites that can degrade the barrier and allow HPV to enter [41]. 

Our results showed that the percentage of NLD cervical samples was slightly shifted when the disease severity increased (Figure 2A). Besides *Lactobacillus* spp., the presence of HPV also affected bacterial diversity (Figure 2B–D). The impact of HPV infection on bacterial diversity was observed in monozygotic twin pairs who had been infected with or without HPV. It was found that the vaginal bacterial diversity in twins with HPV infection was higher than in the HPV-uninfected twin. Moreover, less *Lactobacillus* spp. in HPV-infected patients was demonstrated [42]. This study revealed that bacterial diversity in CIN1 and CIN2/3 were slightly lower than in CIN1 HPV-negative (Figure 2B,C), suggesting that HPV played some role. In the early stages of infection, Hr-HPVs evaded immune response by switching from Th1 to Th2 [43]. Normally, Th1 cells promote the production of proinflammatory cytokines and promote responses against intracellular pathogens such as viruses, whereas Th2 cells produce cytokines against extracellular pathogens such as bacteria. As a result of the increased Th2 response during HPV infection, the bacterial infection may be reduced.

Members of the cervicovaginal microbial community interacted to preserve tissue homeostasis [44]. When this equilibrium is disturbed, a dysbiosis environment develops. There were a number of distinct and common bacterial genera among histological groupings (Figure 3A,B). Numerous BV-involved genera, e.g., *Gardnerella* sp., *Prevotella* sp., and *Atopobium* sp., were discovered in all histological groups of the NLD cohort, indicating CST IV (Figure 3B). Recently, it has been found that *Prevotella* sp. and *Gardnerella* sp. were more abundant in women with persistent HPV16 infection. Overgrowth of *Prevotella* sp. in the vagina has been linked to the development of cervical lesions caused by persistent Hr-HPV infection by influencing host NF-κB and C-myc signaling pathways [45]. In addition, *Atopobium* sp. was one of the bacteria most commonly found in the vagina of HPV-positive patients [46]. Hence, BV-involved genera found in the cervix of our study were correlated with those previously reported in the vagina. Although the dominance of *Lactobacillus* spp. in the vagina and cervix is crucial for cervicovaginal health, some *Lactobacillus* spp., such as the Gram-variable bacteria *L. iners*, may play a pathogenic role [47]. It is capable of producing inerolysin, a pore-forming toxin related to vaginolysin produced by *Gardnerella vaginalis* [47]. In contrast to other *Lactobacillus* spp., *L. iners* was shown to be incapable of producing hydrogen peroxide (H_2_O_2_), which is known to have antibacterial and antiviral activities [48]. Furthermore, CST III vaginal, which was dominated by *L. iners*, had a less acidic pH, but *Lactobacillus* spp. in CSTs I, II, and V were able to produce more acidic pH, which significantly decreased the BV rate [39,49]. Nonetheless, the significance of *L. iners* in illness progression remains unclear, as it was detected in both normal and pathological settings, as demonstrated by the LD group (Figure 3G). *L. iners* predominated in the cervix of Thai women, correlating with a prior study indicating that *L. iners* was the predominant *Lactobacillus* spp. in vaginal swabs from healthy Thai women [37]. In the ubiquity analysis, *Parvimonas* sp. and *Olsenella* sp. were ubiquitous only in NLD, especially in the CIN1 HPV-negative group (100% ubiquity) (Figure 3J,K). *Parvimonas* sp. has been recognized as a genital flora [50]. To date, little is known about *Parvimonas* sp. in terms of vaginal infections or bacterial vaginosis. Interestingly, it has been found that one member of the genus *Parvimonas*, *P. micra*, was linked to the biofilm formation of other bacteria [51]. This suggested that *Parvimonas* sp. might contribute to an infection or bacterial imbalance in the vagina and cervix. In the case of *Olsenella* sp., this bacteria was a member of the vaginal microbiota associated with BV [52]. Together, based on our findings, the high ubiquity of these bacterial genera in the NLD group might be associated with the CST IV community, which consists of various anaerobic bacteria. However, the role of these bacteria in BV and their association with cervical carcinogenesis are under investigation, and further research is needed.

For fungal community, a previous study from Spain demonstrated that the Shannon diversity of fungi in the cervicovaginal area was statistically significantly higher in atypical squamous cell undetermined significant (ASCUS) when compared to the negative for squamous intraepithelial lesion (NSIL) and high-grade squamous intraepithelial lesion (HGSIL) [26]. However, no significant difference in the fungal diversity among cervical groups was shown in this study (Figure 4B–E). Only four fungal OTUs were detected. For example, *C. albicans*, an opportunistic fungal pathogen, colonized 20% of women without causing any clinical manifestations in many healthy women [22]. *Saccharomyces* sp. infection caused vaginitis, but most of the cases were caused by *Saccharomyces cerevisiae* [53]. Interestingly, *Saccharomyces cariocanus* and *Densospora* sp. have never been isolated from the vagina or cervix. The role of *Saccharomyces cariocanus* in vaginitis should be further explored.

Although it has been reported that vaginal bacteria, particularly *Lactobacillus* spp., have a great potential to control cervical illnesses, further research is required [49]. However, our findings indicated that the dominance of *Lactobacillus* spp. did not influence viral diversity (Figure 5A,B). According to the core notion of general virology, the range of hosts for a number of virus types is exceedingly limited and particular [6]. Thus, we continue to concentrate on the diversity of human viral pathogens (Figure 5C,D). We discovered that HPV infection has a significant impact on the decline of human viral diversity. During viral infection, viruses essentially hijack many cellular mechanisms, such as molecular building blocks, such as nucleotides and amino acids, a source of chemical energy, and the cellular protein-synthesizing machinery, in order to support their replication cycle and express their specific proteins [54]. These mechanisms may impede the multiplication of other viruses. The duration of each virus’ lifecycle also influenced viral co-infection, as demonstrated by Vafadar et al.’s model of competitive infection during co-infection [55]. Moreover, host immunity to viral infection plays a crucial role in preventing future viral infections. Viral-infected cells released type I interferon (Type I IFN) to activate the antiviral state in uninfected cells [56], thereby interfering with the emergence of new viruses. Infection with HPV may play all these roles. On the other hand, genital inflammation was previously reported to be associated with a decreasing diversity of cervicovaginal DNA viruses [57]. However, genital inflammation samples were excluded from our study. Taken together, besides genital inflammation, HPV infection was also associated with a reduction of human viral diversity.

Besides HPVs, other human viruses, especially respiratory viruses, e.g., Influenza A virus (H3N2 and H2N2) and Human parainfluenza virus 1, were found in all histological groups (Figure 5E). We hypothesized that these viruses are transmitted to the cervix via specific sexual behaviors, particularly oral sex, but the role of the respiratory viruses in cervical diseases is poorly understood. In our research, many types of TTVs were found in a diverse group of samples. TTV, a member of the family *Anelloviridae* and transmitted through droplets of saliva, is an ubiquitous virus that is found in many types of clinical specimens and is involved in many diseases, e.g., liver diseases, cancer, thalassemia, non-malaria fever, and respiratory illness [58]. Recently, TTV DNA-positive was commonly found in the vagina of healthy pregnancy women [59]. In addition, TTV was also detected in blood of healthy donors [58]. A member of the family *Anelloviridae* was also discovered in the cervix of HIV/HPV co-infected patients [29].

Surprisingly, a few HPV16 reads (relative abundance < 0.1%) were found in the CIN1 HPV-negative group (Figure 5F). At the beginning of the study, we confirmed the absence of HPV DNA in these samples using the REBA HPV test (Molecules and Diagnostics, Wonju, Korea). All were shown negative for HPV DNA. NGS is more sensitive than the conventional diagnostic tests. Interestingly, NGS analysis of all cervical samples collected for this investigation revealed 100% HPV infection. However, the relative abundance of HPV in the CIN1 HPV-negative group was significantly lower when compared to the HPV-positive groups (Figure 5F). Thus, considering this group as either HPV-negative or HPV-positive did not affect the analysis. 

## 5. Conclusions

Our study shed new light on the modifications of the cervical bacterial, fungal, and viral communities in HPV-infected patients with varying degrees of dysplasia severity. We demonstrated that *Lactobacillus* spp. played a significant role in bacterial diversity, while HPV infection played a crucial role in both bacterial and human viral diversity. The correlation of several microorganisms with HPV infection and the severity of dysplasia may be advantageous for the use of microbial communities as diagnostic tools. However, the mode of transmission and function in pathogenesis of many microorganisms, particularly viruses, remain obscure and require further investigation. In addition, our research revealed the presence of non-human viruses in the cervix. Although a small sample size of the CIN1 HPV-negative group was recruited, the statistical analysis of the Shannon index (Figure 2, Figure 4 and Figure 5) obtained from this group showed a normal distribution by the Shapiro–Wilk test (*p* > 0.05), representing a normal population. Thus, all statistically significant analyses in the comparative analysis of the microbiome were reliable. However, according to the limitation of the small sample size, further research is required to confirm and validate these results. More clinical samples should be necessary for further analysis. Additionally, samples of cervical cancer must be included.

## Data Availability

The datasets generated and/or analyzed during the current study are available in the NCBI Sequence Read Archive (SRA) repository under accession number PRJNA766293 (16S rRNA and ITS gene sequencing data, available after publishing) and PRJNA766412 (Metagenomic sequence data generated from Virome Capture Sequencing).

## Supplementary Materials

The following supporting information can be downloaded at: https://www.mdpi.com/article/10.3390/ijerph192214716/s1, Figure S1: The global taxonomic pattern of bacterial abundance for the dominant genera in cervical samples. (A) The abundance of the top 8 genera in the cervix. The samples were divided into 2 groups, i.e., Lactobacilli-dominated (left) and non-lactobacilli-dominated (right). (B) *Lactobacillus* spp. relative abundance (%) in each sample. A blank represents a species that was not detected. (C) The abundant percentage of *Lactobacillus* spp. in LD and NLD groups. Figure S2: Rarefaction curve for Chao1. (A) Bacterial rarefaction measurement in LD and NLD. (B) Chao1 rarefaction curve between LD and NLD pairs in CIN1 HPV-negative, CIN1, and CIN2/3. This index was used to observe the bacterial richness between histological groups. Error bars represent the standard error of the mean (SEM). Figure S3: The global taxonomic pattern of the viral community in cervical samples. The hierarchical clustering tree and heat map of the top 30 viral genera were illustrated. The tree was generated using UPGMA and Euclidean as the algorithm and similarity index, respectively. The color key under the tree represents the cytological and histological characteristics of each cervical sample. From white to blue, color shades represent increasing relative abundance. Figure S4: Ubiquity (%) of the selected viral species among histological groups. The Chi-square test was used to compare the ubiquity between each group. Table S1: Summary of the selected cervical samples. Table S2: The peripheral clinical data of each subject. Table S3: The relative abundance of the selected bacterial taxa in cervical samples. Table S4: The ubiquity (%) of the selected bacterial taxa in cervical samples. Table S5: The abundance and ubiquity (%) of the fungal taxa in cervical samples.
