# Supplementary material for: Cervical Microbiome in Women Infected with HPV16 and High-Risk HPVs
Source: Int J Environ Res Public Health. 2022 Nov 9;19(22):14716. doi: 10.3390/ijerph192214716 (PMC9690271; doi:10.3390/ijerph192214716)
Supplement: Supplementary file 1 [file ijerph-19-14716-s001.zip › Figures S1-S4.pdf]

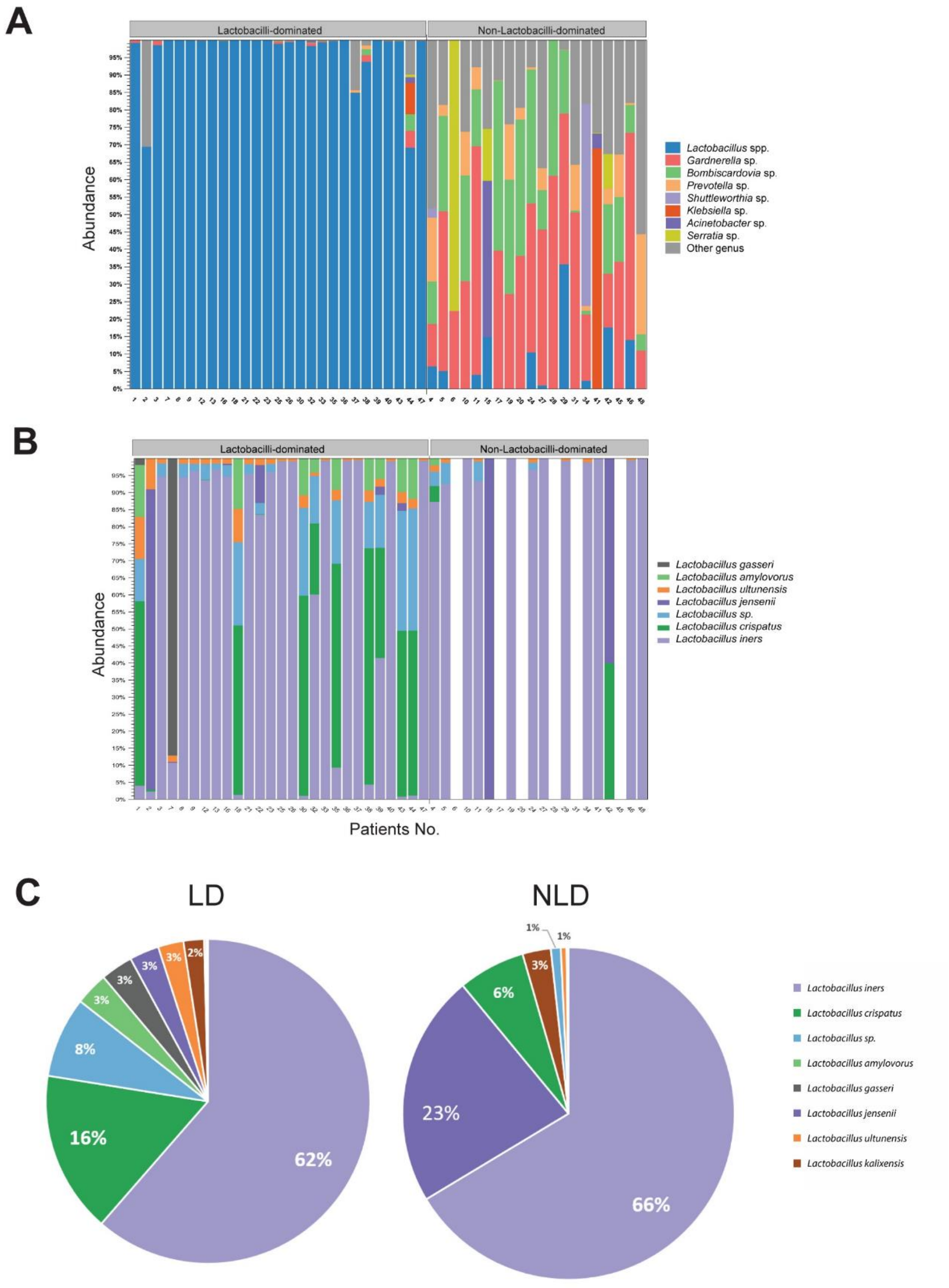

Figure S1 The global taxonomic pattern of bacterial abundance for the dominant genera in cervical. **(A)** The abundance of top 8 genera in cervix. The samples were divided into 2 groups, i.e., Lactobacilli-dominated (left) and Non-lactobacilli dominated (right). **(B)** *Lactobacillus* spp. relative abundance (%) in each sample. A blank represents a species that was not detected. **(C)** The abundant percentage of *Lactobacillus* spp. in LD and NLD groups.

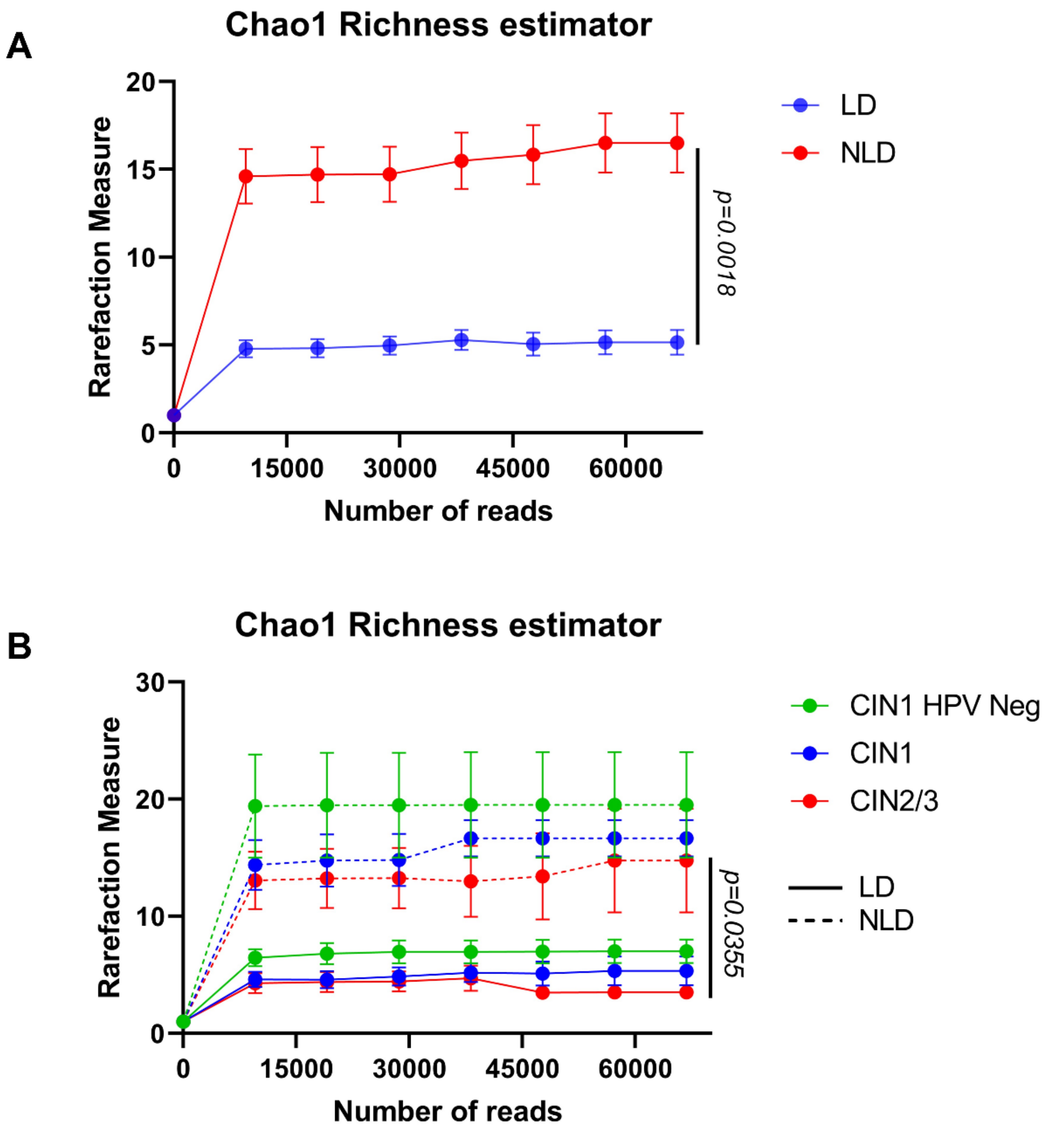

Figure S2 Rarefaction curve for Chao1. **(A)** Bacterial rare fraction measurement in LD and NLD. **(B)** Chao1 rarefaction curve between LD and NLD pairs in CIN1 HPV negative, CIN1, and CIN2/3. This index was used to observe the bacterial richness between histological groups. Error bars represent the standard error of mean (SEM).

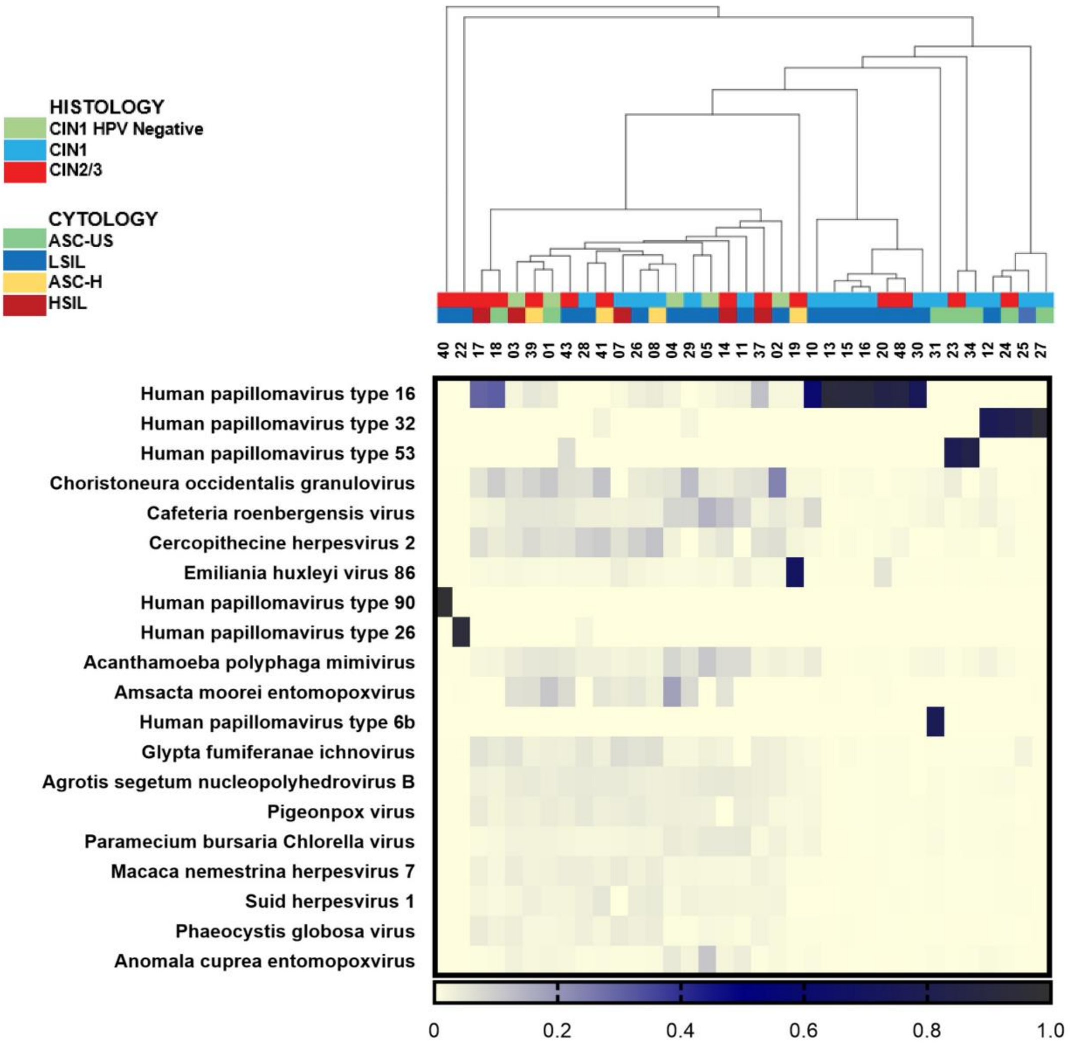

Figure S3 The global taxonomic pattern of the viral community in cervical samples. The hierarchical clustering tree and heat map of the top 30 viral genera were illustrated. The tree was generated using UPGMA and Euclidean as the algorithm and similarity index, respectively. The color key under the tree represents the cytological and histological characteristics of each cervical sample. From white to blue, color shades represented increasing relative abundance.

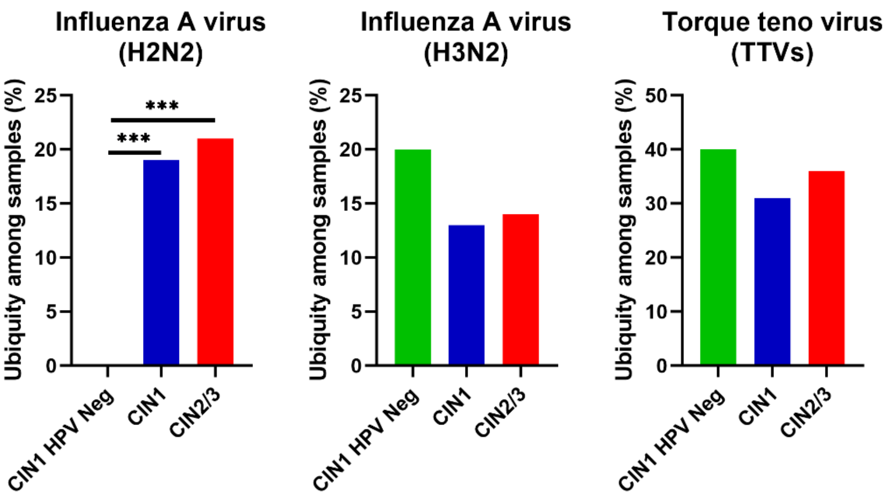

Figure S4 Ubiquity (%) of the selected viral species among histological groups. The Chi-Square test was used to compare the ubiquity between each group.
